# A rare case of neuroendocrine cell tumor mixed with a mucinous component in the ampulla of Vater

**DOI:** 10.1186/s13000-024-01488-z

**Published:** 2024-04-27

**Authors:** Tamotus Sugai, Noriyuki Uesugi, Masamichi Suzuki, Nobuyasu Suzuki, Michitaka Honda, Tsuyoshi Abe, Naoki Yanagawa

**Affiliations:** 1 Diagnostic Pathology Center, Southern Tohoku General Hospital, 7-115, Yatsuyamada, Kooriyama City, Fukushima, 963-8563 Japan; 2 Department of Molecular Diagnostic Pathology, School of Medicine, Iwate Medical University, 2-1-1, Shiwagun, Yahabachou, 028-3695 Japan; 3 Department of Surgery, Southern Tohoku General Hospital, 7-115, Yatsuyamada, Koriyama City, Fukushima, 963-8563 Japan; 4 Department of Minimally Invasive Surgical and Medical Oncology, Fukushima Medical University, 1 Hikarigaoka Fukushima, Fukushima, 960-1295 Japan

**Keywords:** Ampulla of Vater, Grade 2 NET, Mucinous NET, Neuroendocrine cell tumor

## Abstract

A rare case of neuroendocrine cell tumor (NET) having both conventional and mucinous components was reported. Mucinous NET is rarely encountered in the pathological diagnosis of gastrointestinal (GI) tumors. Here we examined the mechanism for transformation of conventional NETs into mucinous NETs. Case presentation: Macroscopic examination revealed a tumor with ulceration in the ampulla of Vater that measured 1.7 cm in its largest diameter. Histologically, the tumor comprised two components: a tubular/ribbon-like feature and small nests floating in a mucinous lake. The tumor nests showed sheet, nest and ribbon-like structures of small cells having eosinophilic cytoplasm as well as small-sized nuclei with dense hyperchromatin. Immunohistochemical analysis showed tumor cells positive for pan-endocrine markers (synaptophysin, CD56, INSM1 and chromogranin). Based on the histological findings, the solid and mucinous components were diagnosed as conventional and mucinous NETs, respectively. Grading was NET G2 based on 12.8% and 13.2% Ki-67-positive cells in the solid and mucinous components, respectively. Immunohistochemically, the mucin phenotype of this tumor was gastric and intestinal. Only the mucinous NET component had cytoplasmic CD10 expression. Examination using a customized gene panel detected only a *DPC4* mutation, which was limited to the mucinous component. Conclusions: Coexistence of conventional and mucinous NETs could provide important insight into evaluating the NET subtype histogenesis. Moreover, molecular alterations including cytoplasmic expression of CD10 and the *DPC4* mutation can contribute to interpretation of tumor pathogenesis.

## Introduction

Gastrointestinal neuroendocrine cell tumors (GI-NETs) are a heterogeneous group of neoplasms arising from neuroendocrine cells, which represent a small fraction of the intestinal crypt [1]. GI-NETs are rare, but their incidence is increasing [2–4]. Gastric NETs (G-NETs) and duodenal NETs (D-NETs) are frequent upper GI-NETs in terms of tumor location [3–5]. D-NETs in particular have attracted increasing attention since duodenal tumors can be treated endoscopically [5, 6]. NETs rarely occur at the ampulla of Vater, and when they do occur at this site they have a different biological behavior [6, 7]. NETs are classified into three distinct subgroups: grade I, II, and III. Grade I (G1)-NETs have a low mitotic rate (< 2 mitoses/2 mm2) and Ki-67 index < 3% [8]. G2 NETs have an intermediate mitotic rate and Ki-67 index, whereas for G3 NETs the mitotic rate and Ki-67 index exceeds 20 mitoses/2 mm2 and > 20%, respectively. NECs (neuroendocrine cell carcinoma) have, by definition, a high mitotic rate (> 20 mitoses/2 mm2) and Ki-67 index > 20% and are classified as either large- or small-cell neuroendocrine carcinoma [8]. This classification is widely used to predict the malignant behavior of NETs [8].

Patients with NETs in the ampulla of Vater were recently shown to have worse overall survival (OS) than those having NETs in the duodenum [3, 4]. The reasons for this poor prognosis are unclear. Here we describe a rare case with histological features of mucinous NET, characterized by cells floating within a mucinous lake, mixed with NET G2.

## Case presentation

The patient was a Japanese woman in her 70 s who had no physical syndrome. She visited our hospital for liver dysfunction but had no jaundice. Computed tomography (CT) and endoscopic retrograde cholangiopancreatography (ERCP) revealed no finding at the time of her hospital visit. Biannual follow-up CTs were performed. Two years after the initial visit, CT and ERCP were performed again due to mild fever and left abdominal pain. The ERCP showed an ~ 20-mm mass in the ampulla of Vater with an ulcer and bleeding. A biopsy was performed.

**Fig. 1:**
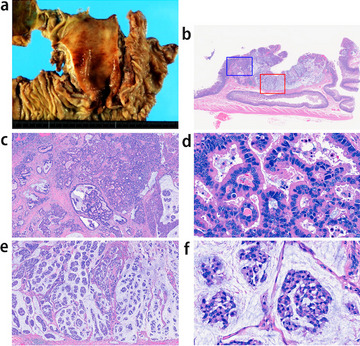
Macroscopic and histological figures of the tumor sample. **a** Macroscopic features; **b** Lupe figure of the tumor; Examination of conventional (**c**, **d**) and mucinous (**e**, **f**) NET components at low- and higher-power magnification

A pancreaticoduodenectomy was performed after pathological findings of the endoscopic biopsy of the ampulla of Vater suggested the presence of a malignant tumor. Macroscopic examination revealed a tumor in the duodenal papilla measuring 1.7 cm in its largest diameter. Irregular nodules, bleeding and ulceration consistent with endoscopic findings were seen (Fig. 1-a).

**Fig. 2:**
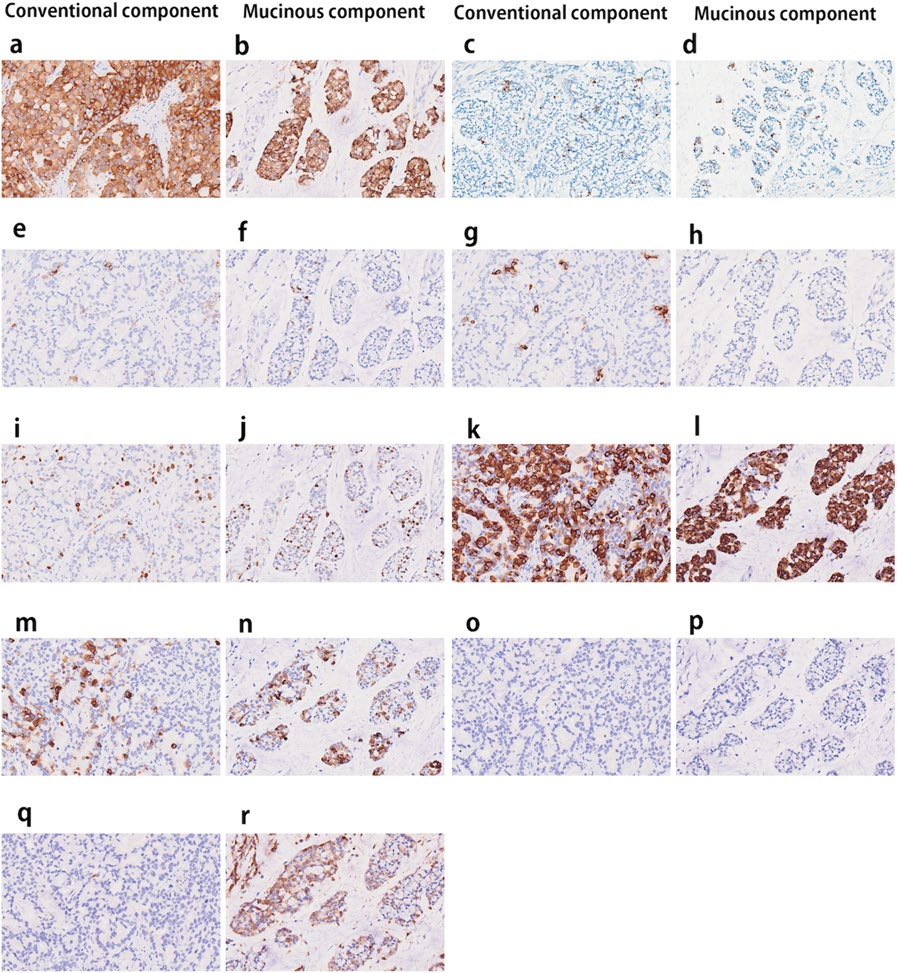
Immunohistochemical findings of the tumor sample. Diffuse positive staining of synaptophysin in the (**a**) conventional and (**b**) mucinous NET component. Scattered positive staining of INSM1 in the (**c**) conventional and (**d**) mucinous NET component. Scattered positive staining of CD56 in the (**e**) conventional and (**f**) mucinous NET component. Scattered positive staining of chromogranin in the (**g**) conventional and (**h**) mucinous NET component. Ki-67 positive ratio in (**i**) conventional and (**j**) mucinous NET is 3.4%. Diffuse positive MUC2 staining in (**k**) conventional and (**l**) mucinous NET. Focal positive staining of MUC5AC in (**m**) conventional and (**n**) mucinous NET. Negative staining of MUC6 in (**o**) conventional and (**p**) mucinous NET. Negative CD10 expression in (**q**) conventional NET and (**r**) positive CD10 cytoplasmic staining in mucinous NET

Histologically, the tumor had two components including a solid nest and mucinous lake formation (Fig. 1-b). The solid nest was characterized as sheets and nests with a ribbon like-structure of small cells having eosinophilic cytoplasm and small-sized nuclei with dense hyperchromatin and little mucus (Fig. 1-c, d). Such nests did not show trabecular or well-differentiated tubular features and also lacked goblet and Paneth-like cells. In addition, mucous cells scattered within the nests was found in the present case. The mucinous lake formation resembled mucinous carcinoma (Fig. 1-e) and had nests of tumor cells surrounded by extracellular mucin; the cell nests floated within an extra-cellular mucin lake (Fig. 1-f) that histologically resembled those of the solid component (Fig. 1-e and -f). However, pleomorphic nuclear features and well- to moderately-differentiated tubular formations that are typically found in duodenal adenocarcinoma were not seen in the current case. On the basis of the findings, the nests of the two components were suggestive of NET. We thus considered that the tubular/ribbon-like nests and the nests within the mucinous lake were NETs, specifically, conventional and mucinous NETs, respectively. Next, we examined immunohistochemical endocrine makers including synaptophysin, INSM1, CD56 and chromogranin to determine the NET’ characteristic. Immunohistochemical analysis was positive for several pan-endocrine markers (synaptophysin, INSM1, CD56 and chromogranin) (Fig. 2-a-h; Table 1). Although INSM1, CD56 and chromogranin positivity was scattered (Fig. 2c–h), expression of synaptophysin was diffuse positive (Fig. 1a and -b). The sample was negative for the functional markers including somatostatin, insulin, glucagon, gastrin, and serotonin. Fourteen lymph nodes were identified and showed no tumor involvement. The final diagnosis of the two tumor components was neuroendocrine cell tumor, NET, grade 2, of the duodenal papilla, based on auto-analyzer measurements at hot spot regions that showed a 12.8% and 13.2% Ki-67 index in the solid nest and mucinous components, respectively. Hot spots in the solid nest and mucinous components had mitotic counts of 3 and 5, respectively (Fig. 2-i and j). We also examined immunohistochemical expression of mucin markers (MUC2, MUC5AC and MUC6) and CD10. Both components had diffuse positive MUC2 expression (Fig. 2-k and -l) and focal MUC5AC expression (Fig. 2-m and -n). No MUC6 staining was detected in either component (Fig. 2-o and -p). The mucin phenotype was determined to be mixed gastric and intestinal (GI phenotype). The mucinous component was positive for cytoplasmic CD10 expression, but the conventional component was negative (Fig. 2-q and -r). Separate examination of gene mutations in both components using a customized gene panel containing 28 genes (APC, BRAF, TP53, CDKN2A, MET, ATM, MLH-1, PMS2, HRAS, AXIN2, BAX, DCC, MSH2, POLE, RNF43, PTEN, EPCAM, MSH6, BUB1B, RHOA, KRAS, NRAS, SMAD4, CDK4, PIK3CA, STK11, TGFBR2, and EGFR) detected only a DPC4 mutation that was limited to the mucinous component (R361H (cGc > cAc).

**Table 1:** Immunohistochemical expression of examined markers

	Solid nest component (Conventional)	Mucinous component
Synaptophysin	2 +	2 +
Chromogranin	scattered positive	scattered positive
CD56	scattered positive	scattered positive
Ki-67	12.8%	13.2%
p53	negative	negative
Insulin	negative	negative
Glucagon	negative	negative
Somatostatin	negative	negative
Gastrin	negative	negative
Serotonin	negative	negative
MUC2	positive	positive
MUC5AC	focal positive	focal positive
MUC6	negative	negative
CD10	negative	cytoplasmic expression

## Discussion

Goblet cell carcinoid (GCC) and conventional adenocarcinoma with endocrine cell differentiation are important factors for the differential diagnosis of disease in this patient and can be distinguished based on histological, immunohistochemical and molecular findings [9, 10]. GCC is a histologically amphicrine neoplasm containing goblet-like mucinous cells with varying numbers of endocrine cells and Paneth-like cells that typically have a tubular or clustered (or trabecular) arrangement [9]. However, the current case did not have such characteristic cytological findings with neither goblet-like nor Paneth-like cells found. Moreover, this case did not have the typical architecture of the GCC tumor cell nests and instead had medium- to large-sized nests rather than small, trabecular nests that are frequently found in GCC [9]. Based on these histological findings, GCC can likely be excluded from the histological diagnosis [9]. Second, conventional adenocarcinoma with endocrine cell differentiation is histologically characterized by pleomorphic nuclear features, and well- to moderately-differentiated tubular formations, but these features, which are typically found in duodenal adenocarcinoma, were not seen in the current case [11, 12]. In particular, nuclear pleomorphism is an important finding for differential diagnosis between duodenal adenocarcinoma and duodenal NET [11, 12]. However, the current case lacked nuclear pleomorphism and a high mitotic count that is generally seen for duodenal adenocarcinoma. In addition, synaptophysin in both tumor components of the current case, which supports the diagnosis of NET since in adenocarcinoma with endocrine cell differentiation endocrine cells are scattered within cancer nests. The diagnosis for the current case is further supported by the finding that only a *DPC4* mutation was detected. In contrast, the specific mutational spectrum of duodenal adenocarcinoma frequently includes mutations in *TP53* and *KRAS*, which are rare in NETs [13]. These findings suggest that conventional NET can transform into mucinous NET that may arise from mucous cells scattered within the nests. Indeed, a case of ductal carcinoma of the breast was reported to have endocrine differentiation resulting in mucinous NEC [14]. However, the present case differs in the coexistence of conventional and mucinous NETs. To our knowledge, this is the first case of conventional NET that progressed into mucinous NET in the ampulla of Vater.

NET grading as G1, G2 and G3 describes the aggressive or malignant nature of a tumor [8]. The tumor in the present case was G2 based on its 3.8% Ki-67 positive rate and its mitotic count. Previous studies indicated that the prognosis of ampulla of Vater NET is poorer than that of duodenal NET [4, 15]. However, the prognosis of G2 NET in general is currently unclear since there were few studies describing NET grading including G2 before the proposal of the World Health Organization that was published in 2010 [13].

In immunohistochemical analysis of gastric mucin markers and CD10, the tumor had a GI phenotype suggestive of differentiation toward a gastric and intestinal phenotype [16]. The staining pattern is supported by the previous finding that a GI phenotype is common in rectal NET [16]. Here cytoplasmic expression of CD10 was detected only in mucinous NET. Cytoplasmic expression of CD10 correlates with tumor aggressiveness and increased metastatic potential, especially in colorectal cancer [17, 18]. The clinicopathological significance of cytoplasmic CD10 expression in NETs should be elucidated in the future.

We detected no mutations in the tumor in an examination using a customized panel of 28 genes that are closely associated with GI tumor carcinogenesis. This finding suggests that mutations that drive GI cancer may be less relevant to NET G2. Meanwhile, TP53 and *DPC4* mutations that contribute to GI tumorigenesis [19] rarely occur in well-differentiated endocrine tumors [20]. Here we found no *TP53* mutation, but a *DPC4* mutation was detected in the mucinous NET component indicating that DPC4 could promote tumorigenesis in some NETs [21].

In summary, this tumor has both conventional NET G2 and a mucinous component termed mucinous NET. This NET variant is a rare tumor in duodenal NET, especially in the ampulla of Vater, and the prognosis may be assumed to be poor. Additional cases are needed to fully characterize the pathogenesis of this variant. Finally, only the mucinous component had cytoplasmic CD10 expression and *DPC4* mutation that both may be associated with progression from conventional to mucinous NET.

## Data Availability

No datasets were generated or analysed during the current study.
